# Death after discharge: prognostic model of 1-year mortality in traumatic brain injury patients undergoing decompressive craniectomy

**DOI:** 10.1186/s41016-021-00242-4

**Published:** 2021-04-21

**Authors:** Wenxing Cui, Shunnan Ge, Yingwu Shi, Xun Wu, Jianing Luo, Haixiao Lui, Gang Zhu, Hao Guo, Dayun Feng, Yan Qu

**Affiliations:** Department of Neurosurgery, Tangdu Hospital, No. 569 Xin Si Road, Xi’an, 710038 Shaanxi Province China

**Keywords:** Decompressive craniectomy, Traumatic brain injury, One-year mortality, Prognostic model, Random forest

## Abstract

**Background:**

Despite advances in decompressive craniectomy (DC) for the treatment of traumatic brain injury (TBI), these patients are at risk of having a poor long-term prognosis. The aim of this study was to predict 1-year mortality in TBI patients undergoing DC using logistic regression and random tree models.

**Methods:**

This was a retrospective analysis of TBI patients undergoing DC from January 1, 2015, to April 25, 2019. Patient demographic characteristics, biochemical tests, and intraoperative factors were collected. One-year mortality prognostic models were developed using multivariate logistic regression and random tree algorithms. The overall accuracy, sensitivity, specificity, and area under the receiver operating characteristic curves (AUCs) were used to evaluate model performance.

**Results:**

Of the 230 patients, 70 (30.4%) died within 1 year. Older age (OR, 1.066; 95% CI, 1.045–1.087; *P* < 0.001), higher Glasgow Coma Score (GCS) (OR, 0.737; 95% CI, 0.660–0.824; *P* < 0.001), higher d-dimer (OR, 1.005; 95% CI, 1.001–1.009; *P* = 0.015), coagulopathy (OR, 2.965; 95% CI, 1.808–4.864; *P* < 0.001), hypotension (OR, 3.862; 95% CI, 2.176–6.855; *P* < 0.001), and completely effaced basal cisterns (OR, 3.766; 95% CI, 2.255–6.290; *P* < 0.001) were independent predictors of 1-year mortality. Random forest demonstrated better performance for 1-year mortality prediction, which achieved an overall accuracy of 0.810, sensitivity of 0.833, specificity of 0.800, and AUC of 0.830 on the testing data compared to the logistic regression model.

**Conclusions:**

The random forest model showed relatively good predictive performance for 1-year mortality in TBI patients undergoing DC. Further external tests are required to verify our prognostic model.

## Background

TBI is a common cause of death and disability worldwide affecting all age groups [1]. Decompressive craniectomy (DC), surgically removing a component of the skull, has been performed in TBI patients for many years [2, 3], especially those with high intracranial pressure (ICP) [4], which can effectively increase cerebral perfusion pressure. Two randomized clinical trials have been conducted, the DECRA [5] and RESCUEicp [6] trials, which focused on the prognosis of TBI patients after DC. DECRA demonstrated that early bifronto-temporo-parietal DC decreased the length of stay in the ICU but was associated with unfavorable outcomes; RESCUEicp reported that DC in patients with TBI and refractory intracranial hypertension led to lower mortality.

In previous research, short-term outcome predictive scoring models, discharge status [7] and 30-day mortality [8], were well established in TBI patients with DC. However, a long-term mortality prediction model is a crucial issue that has not received sufficient attention. Despite several studies on long-term outcomes, such as predictors of 1-year mortality in older brain-injured patients [9] and functional outcomes from 3 to 24 months following severe brain injury [10], the study population was not specifically focused on patients after DC. It is necessary to identify the predictors of long-term mortality in TBI patients after DC to gain a better understanding of the progression of the disease, contributing to better daily care and improvements in the quality of life of patients.

Machine learning has been widely used in disease diagnosis and prognosis prediction [11, 12]. For example, machine learning was applied to predict in-hospital morbidity and mortality after TBI, which demonstrated relatively good predictive performance [13]. To date, the use of machine learning techniques to predict the long-term prognosis of TBI patients after DC has rarely been explored. Thus, the purpose of this study was to develop prognostic models to predict the 1-year mortality of TBI patients undergoing DC by using logistic regression and random tree models.

## Methods

### Patient population

This retrospective study was approved by the Ethics Committee of Tangdu Hospital, Fourth Military Medical University. We reviewed 947 consecutive TBI patients treated at Tangdu Hospital from January 1, 2015, to April 25, 2019. Our main inclusion criterion covered patients who underwent DC with a history of TBI. The main indications and approach were described in previous studies [14, 15]. The exclusion criteria were as follows: (1) an interval from injury to admission of more than 24 h; (2) death in the hospital; (3) other severe systemic diseases, such as malignant tumors, cirrhosis, and uremia; and (4) loss to follow-up.

### Variables and data collection

The following data were extracted from the registry database by five study nurses: patient demographic characteristics; Glasgow Coma Score (GCS) score in admission; biochemical tests including aPTT, INR, platelet counts, D-dimer, fibrinogen, glucose, red blood cell, and neutrophil/lymphocyte ratio (NLR); initial CT scan characteristics including contusion volume, subarachnoid hemorrhage (Fisher scales), midline shift, and basal cistern status; perioperative bleeding; and worsening neurologic condition, including mechanical ventilation, tracheotomy, deep venous thrombosis, and hypotension that needed noradrenaline to correct. INR, aPTT, and platelet counts were used to define traumatic coagulopathy, according to a previous study [16], aimed at simplifying the prognostic model. Coagulopathy was defined as an aPTT > 36 s and/or a PT in INR > 1.2 and/or a platelet count < 100 × 10^9^ per liter.

### Prognostic model

Of the 230 patients, 172 patients (75%) were randomly selected for training, and the remaining 58 patients (25%) were selected for testing. The random seed was set as 66,511. The ratio of non-survivors to survivors was 1:2, so synthetic minority oversampling technique (SMOTE) was used to balance the training data. We conducted a performance comparison of the logistic regression and random tree models. The 10-fold cross-validation, repeated three times, was performed by using the original 75% of the data treated as training data. Features were selected by using the univariate logistic regression method. Hyperparameter optimization was achieved by the grid search method. Three parameters were determined for the random tree model: “Gini” impurity criterion, mtry = 4, and tree = 100. We determined the number of mtry by grid optimization algorithm. An open-source programming language R 3.6.1 and an efficient machine learning tool GraphLab Create were used for machine learning coding.

We compared the predictive performance of the logistic regression and random tree models according to accuracy, sensitivity, specificity, and AUC. The AUCs of the two models on the testing data are the results of back-substituting the training set to the models. The definitions of accuracy, sensitivity, and specificity were described in a previous study [17].

### Statistical analysis

Categorical variables are expressed as frequencies (percentages), and continuous variables with skewed distributions are presented as medians and interquartile ranges (IQRs). Univariate logistic regression was used to identify significant predictive variables (*P* < 0.05), which were entered into the multivariate regression by using the forward LR method to determine the independent risk factors for 1-year mortality. A nomogram model was developed to predict the probability of 1-year mortality. All data were analyzed with the statistical software SPSS 20.0 (IBM, New York, NY).

## Results

### Patient population

**Fig. 1:**
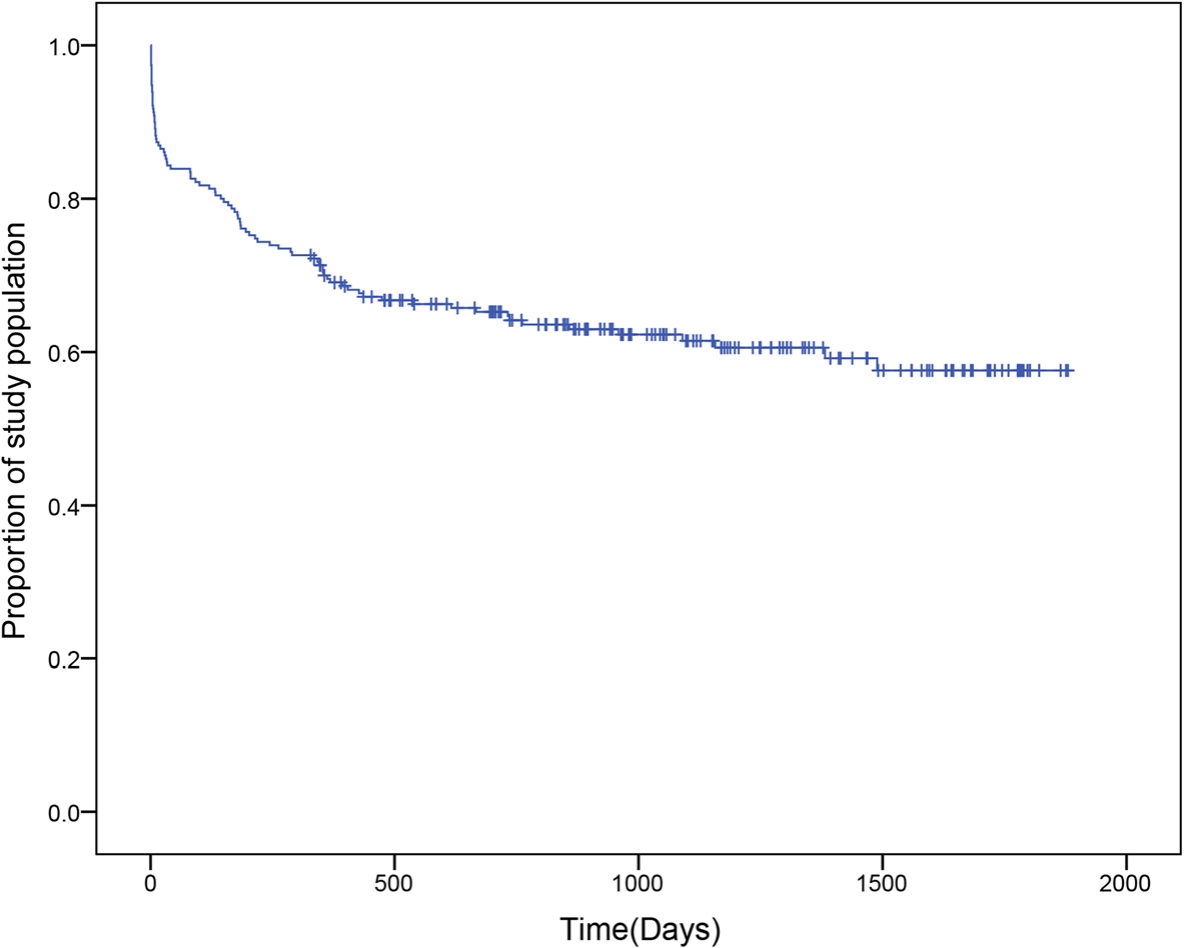
Kaplan-Meier survival curves for TBI patients undergoing DC

Of the 947 patients treated in this research center during the study period, 306 patients who underwent DC were identified. According to our inclusion and exclusion criteria, 230 patients were enrolled. By February 26, 2020, 89 (38.7%) patients had died, and 141 (61.3%) had survived. The survival analysis showed that the 3-month survival rate was 0.826, the 6-month survival rate was 0.774, the 1-year survival rate was 0.695, and the 3-year survival rate was 0.623 (Fig. 1).

The patient demographic and clinical characteristics are summarized in Table 1. Seventy patients (30.4%) died within 1 year of undergoing DC. The median age was 59 years (IQR, 50–65), and 16 (22.86%) patients were female. In this study, the most frequent mechanism of injury was motor vehicle accidents (114 patients; 49.57%); falling was also common (82 patients; 36.65%). The median GCS score at admission was 4.5 (IQR, 3–7). The total biochemical tests were as follows: Fibrinogen was 1.86 g/L (IQR, 1.39–2.27). D-dimer was 42.56 mg/mL (IQR, 18.47–98.10). Fifty-one (22.17%) patients had coagulopathy disorders. Abnormal glucose (>8.33 mmol/L) and red blood cells (man >5.5×10^12^/L or <4.0×10^12^/L, woman >5.0×10^12^/L or <3.5×10^12^/L) were shown in 159 (69.13%) patients and 56 (24.56%) patients, respectively. The N/L ratio was 14.55 (IQR, 8.29–24.57). In the initial CT scan characteristics, the contusion volume was 15.99 cm^3^ (IQR, 0–40.82), and subarachnoid hemorrhage (Fisher scale) was 2 (0–3) in total. Midline shift and completely effaced basal cisterns were noted in 97 (42.17%) and 40 (17.39%) patients, respectively. Perioperative bleeding was more than 750 mL shown in 178 (77.39%) patients. Worsening neurologic conditions are listed as follows: mechanical ventilation, tracheotomy, deep venous thrombosis, and hypotension requiring noradrenaline were noted in 84 (36.52%), 102 (44.35%), 22 (9.57%), and 39 (16.96%) patients, respectively.

**Table 1:** Univariate logistic regression analysis of 1-year mortality

	Total (*n*=230)	Survivor (*n*=160)	Non-survivor (*n*=70)	Odds ratio 95% CI	*P* value
Age, years	52.50 (42.00–60.00)	50.50 (38.00–58.00)	59.00 (50.00–65.00)	1.050 (1.025–1.076)	**<0.001**
Female	58 (25.22%)	42 (26.25%)	16 (22.86%)	0.832 (0.430–1.610)	0.586
Mechanism of injury				0.855 (0.632, 1.158)	0.312
Motor vehicle accident	114 (49.57%)	73 (45.63%)	41 (58.57%)		
Fall	82 (36.65%)	63 (39.38%)	19 (27.14%)		
Strike	7 (3.04%)	5 (3.13%)	2 (2.86%)		
Others	27 (11.74%)	19 (11.88%)	8 (11.43%)		
GCS score	6.00 (4.00–8.00)	7.00 (4.00–8.00)	4.50 (3.00–7.00)	0.798 (0.704–0.904)	**<0.001**
Fg (g/L)	1.86 (1.39–2.27)	1.86 (1.44–2.37)	1.81 (1.23–2.18)	0.769 (0.544–1.087)	0.137
D-dimer (mg/L)	42.56 (18.47–98.10)	37.26 (16.61–76.46)	75.17 (31.41–131.40)	1.009 (1.005–1.014)	**<0.001**
Coagulopathy	51 (22.17%)	29 (18.13%)	22 (31.43%)	2.070 (1.086–3.947)	**0.027**
GLU >8.33mmol/L	159 (69.13%)	110 (68.75%)	49 (70.00%)	1.061 (0.576–1.954)	0.85
RBC (man >5.5×10^12^/L or <4.0×10^12^/L, woman >5.0 ×10^12^/L or <3.5×10^12^/L)	56 (24.56%)	40 (25.32%)	16 (22.86%)	0.874 (0.450–1.697)	0.691
N/L ratio	14.55 (8.29–24.57)	14.12 (8.59–22.09)	14.85 (7.38–27.08)	1.008 (0.987–1.030)	0.448
Midline shift >0	97 (42.17%)	73 (45.63%)	24 (34.29%)	0.622 (0.347, 1.114)	0.109
Completely effaced basal cisterns	40 (17.39%)	20 (12.50%)	20 (28.57%)	2.800 (1.392–5.632)	**0.004**
Subarachnoid hemorrhage (Fisher scales)	2.00 (0.00–3.00)	2.00 (0.50–3.00)	2.00 (0.00–3.00)	0.929 (0.759–1.138)	0.478
Contusion volume (cm^3^)	15.99 (0.00–40.82)	14.48 (0.00–44.20)	20.13 (0.00–38.01)	0.999 (0.993–1.005)	0.822
Perioperative bleeding >750 mL	178 (77.39%)	118 (73.75%)	60 (85.71%)	2.136 (1.002–4.550)	**0.049**
Mechanical ventilation	84 (36.52%)	53 (33.13%)	31 (44.29%)	1.605 (0.903–2.852)	0.107
Tracheotomy	102 (44.35%)	70 (43.75%)	32 (45.71%)	1.083 (0.616–1.904)	0.783
Deep venous thrombosis	22 (9.57%)	15 (9.38%)	7 (10.00%)	1.074 (0.418–2.762)	0.882
Use of noradrenaline to treat hypotension	39 (16.96%)	21 (13.13%)	18 (25.71%)	2.291 (1.131–4.640)	**0.021**

### Prognostic factors predicting 1-year mortality in the univariate and multivariate analyses

Univariate analyses of the relationship between clinical variables and 1-year mortality are shown in Table 1. Age (*P* < 0.001), lower GCS (*P* < 0.001), higher D-dimer (*P* < 0.001), coagulopathy (*P*= 0.027), hypotension (*P*= 0.021), completely effaced basal cisterns (*P*= 0.004), and perioperative bleeding > 750 mL (*P* = 0.049) were associated with 1-year mortality. Table 2 lists the results of the multivariate regression analysis to predict 1-year mortality. Older age (*P* < 0.001), lower GCS (*P* < 0.001), higher D-dimer (*P* = 0.015), coagulopathy (*P* < 0.001), hypotension (*P* < 0.001), and completely effaced basal cisterns (*P*= 0.004) were independent predictors of 1-year mortality.

**Table 2:** Multivariate logistic regression analysis of 1-year mortality

Variable	*β* coefficient	Odds ratio	95% CI	*P* value
Age	0.064±0.010	1.066	1.045–1.087	<0.001
GCS score	−0.305±0.057	0.737	0.660–0.824	<0.001
D-dimer	0.005±0.002	1.005	1.001–1.009	0.015
Coagulopathy				<0.001
No		1.0 (referent)		
Yes	1.087±0.253	2.965	1.808–4.864	
Use of noradrenaline to treat hypotension				<0.001
No		1.0 (referent)		
Yes	1.351±0.293	3.862	2.176–6.855	
Completely effaced basal cisterns				<0.001
Normal		1.0 (referent)		
Abnormal	1.326±0.262	3.766	2.255–6.290	

**Fig. 2:**
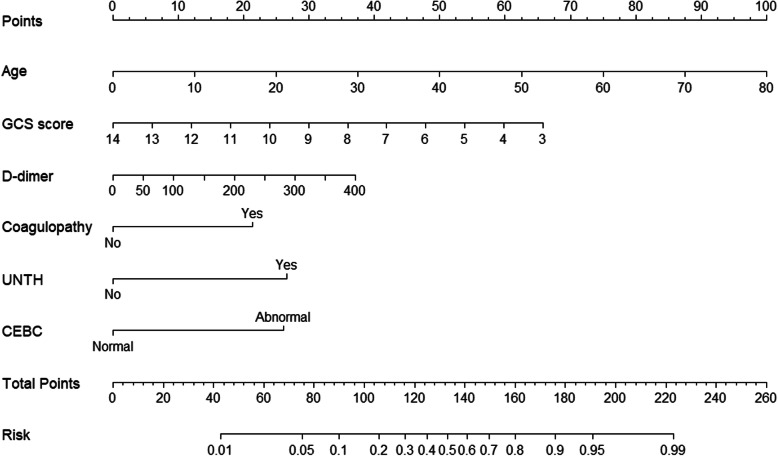
Nomogram for predicting 1-year mortality unfavorable outcome in TBI patients undergoing DC. The total points are calculated as the sum of the individual scores of 6 variables included in the nomogram. UNTH, use of noradrenaline to treat hypotension; CEBC, completely effaced basal cisterns

Next, we incorporated all independent predictors identified in multivariate regression analysis to create nomograms, as shown in Fig. 2. The nomogram was constructed by setting a score to each parameter with a point ranging from 0 to 100. Summing the points arranged for each predictor yields the total score, which is ultimately converted into an individual probability of 1-year mortality (from 1 to 99%). Based on the condition of the patients, this nomogram can predict 1-year mortality both simply and intuitively.

### Prediction performance of logistic regression and random tree models

**Fig. 3:**
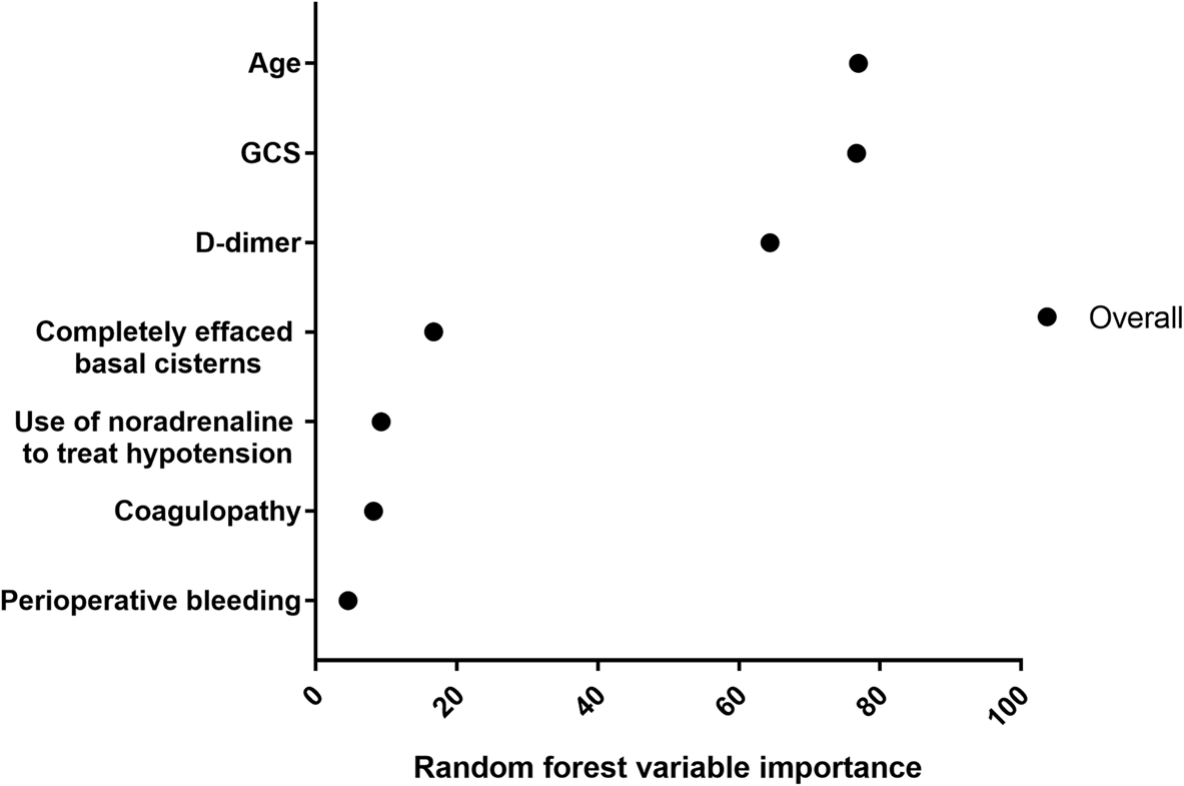
Variable importance measures for each predictor of 1-year mortality derived from random forest

To identify the importance of each predictor for 1-year mortality, we chose the feature selection method by applying the random forest algorithm. Three parameters were identified as the most important for predicting 1-year mortality: age, GCS, and D-dimer (Fig. 3). Interestingly, perioperative bleeding was identified as the last associated factor for 1-year mortality.

**Fig. 4:**
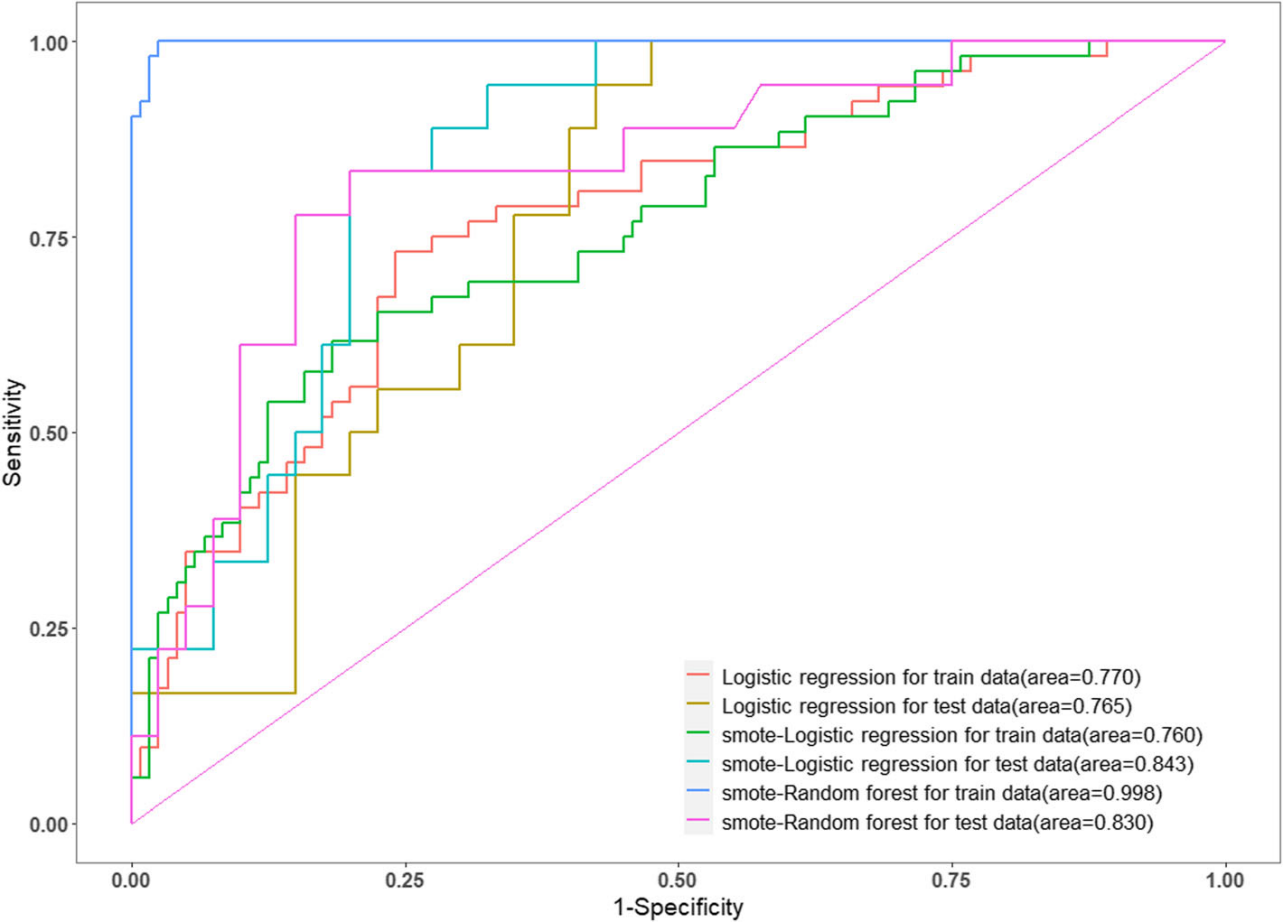
Receiver operating characteristic curves for 1-year mortality prediction in the training sample and test sample

Their ROC and AUC were calculated to evaluate their discriminative ability (Fig. 4). To evaluate the prediction performance of the logistic regression and random tree models, 10-fold cross-validation was performed on the training data (Table 3). Before SMOTE was used to balance the training data, we developed the logistic regression model. On the training data, it achieved an overall accuracy of 0.750, sensitivity of 0.731, specificity of 0.758, and AUC of 0.770 at the optimal cutoff point (0.307); on the testing data, it achieved an overall accuracy of 0.672, sensitivity of 1.000, specificity of 0.525, and AUC of 0.765 when at optimal cutoff point (0.195). Next, we balanced the training data by using SMOTE. The logistic regression model achieved an overall accuracy of 0.756, sensitivity of 0.615, specificity of 0.817, and AUC of 0.760 on the training data and an overall accuracy of 0.741, sensitivity of 0.889, specificity of 0.675, and AUC of 0.843 on the testing data at the optimal cutoff point. The random forest model achieved an overall accuracy of 0.983, sensitivity of 1.000, specificity of 0.975, and AUC of 0.998 on the training data and an overall accuracy of 0.810, sensitivity of 0.833, specificity of 0.800, and AUC of 0.830 on the testing data at the optimal cutoff point. The accuracy, sensitivity, specificity, and AUC at the cutoff point (0.5) of the three prognostic models are shown in Table 3.

**Table 3:** The 1-year mortality prediction performance of logistic regression models and random forest models for the training sample and test sample

		Cutoff	Accuracy	Sensitivity	Specificity	AUC
Logistic regression	Train data	0.5	0.744	0.346	0.917	0.770
		Optimal (0.307)	0.750	0.731	0.758	
	Test data	0.5	0.655	0.167	0.875	0.765
		Optimal (0.195)	0.672	1.000	0.525	
SMOTE logistic regression	Train data	0.5	0.686	0.692	0.683	0.760
		Optimal (0.641)	0.756	0.615	0.817	
	Test data	0.5	0.741	0.722	0.750	0.843
		Optimal (0.405)	0.741	0.889	0.675	
SMOTE random forest	Train data	0.5	0.959	1.000	0.942	0.998
		Optimal (0.596)	0.983	1.000	0.975	
	Test data	0.5	0.828	0.778	0.850	0.830
		Optimal (0.487)	0.810	0.833	0.800	

## Discussion

In our study, we found that older age, lower GCS, higher D-dimer, coagulopathy, hypotension, and completely effaced basal cisterns were independent predictors of 1-year mortality in patients with TBI after DC. Compared to the logistic regression model, the random tree model presented a better performance on the training data with respect to accuracy, sensitivity, specificity, and AUC (regardless of whether the cutoff point was 0.5 or the optimal point). So did the random tree model in regard to accuracy, sensitivity, and specificity on the testing data when the cutoff point was 0.5. Although the sensitivity of the random tree model was inferior to that of the logistic regression model on the testing data at the optimal cutoff point, the accuracy and specificity of the random tree model were superior. The AUCs of the two models are similar on the testing data. Overall, this finding suggests that the random tree is a valuable and accurate model to predict 1-year mortality in TBI patients after DC. Additionally, our study chose the time of 1-year mortality based on the survival analysis of TBI patients undergoing DC, which showed that the mortality rate within 1 year after discharge was very high. Since we turned the spotlight on the long-term outcomes of TBI patients, patients who died in the hospital were excluded. The predictors of inpatient death and postdischarge mortality were disparate, as shown by a previous study [9]. Thus, our study on 1-year mortality, which excluded patients who died in the hospital, could show better predictive performance to some extent.

Age and GCS, which were already found to be important predictors of TBI, were also confirmed in our study [13, 18]. Tian et al. [7] identified that age was one of the independent risk factors for discharge status after DC, and Tang et al. [8] also observed that age was a risk factor for 30-day mortality after DC. Combined with our research, older age is considered a risk factor for both short-term and long-term outcomes of TBI patients after DC. Older people tend to suffer from more basic diseases than young people, and the rehabilitation of the body is poorer after TBI. GCS, which was similar to age, was also a powerful predictor for the outcomes of TBI after DC [8, 19]. D-dimer, a degradation product of fibrinogen, reflects the level of fibrinolysis in the body. Many studies found that higher d-dimer at admission was associated with a higher risk of progressive hemorrhagic injury [20, 21], while a meta-analysis of the prognostic role of d-dimer level on admission in TBI patients found no significant relationship between d-dimer and the risk of poor functional outcome at 3 months [22]. In our study, higher D-dimer was one of the predictors for 1-year mortality after DC. In our opinion, the prognostic role of d-dimer may be related to the study population and specific outcomes. Secondary coagulopathy after TBI is considered an important factor for unfavorable outcomes [23, 24], and our results also confirmed this finding. TBI-induced coagulopathy is very common, ranging from 7 to 54% [25, 26]. Coagulopathy generated by TBI is a systemic manifestation of local injury [27]. Procoagulant vesicles (including tissue factors, cardiolipin, vWF) from damaged brain tissue are released into the systemic circulation [28–30], disrupting the balance between coagulation and anticoagulation. This distinct pathogenetic pathway has attracted increasing attention, and how to intervene in this process is crucial for the prognosis of TBI patients. Hypotension was another risk factor for 1-year mortality. The prognostic role of hypotension in TBI is poorly elaborated. Tang et al. [8] found that intraoperative hypotension was associated with 30-day mortality in TBI patients after DC. In our study, we recorded the incidence of hypotension throughout the course of the disease. Additionally, noradrenaline was injected to maintain the vital signs in patients with hypotension. Our study suggests that hypotension is a crucial predictor of long-term prognosis that should not be ignored in TBI patients after DC. However, some more specific questions between hypotension and the outcome of TBI need to be addressed. For example, it is unclear whether the course of hypotension in patients with TBI is associated with patient outcomes. The risk factors underlying hypotension in TBI need to be explored further. Completely effaced basal cistern status, which represents severely elevated ICP, was found to be an important predictor of outcome in a previous study [8, 31]. Basal cistern effacement is closely associated with pupillary reactivity midline shift. Thus, it can represent a uniquely useful neuroimaging characteristic to guide intervention in TBI [32]. Our study focused on developing prognostic models of predicting the 1-year mortality of TBI patients undergoing DC. The TBI patients who underwent DC or not were different in some characteristics. And further studies are needed to explore these differences.

Many studies on TBI have been conducted using modern machine learning algorithms owing to their good prediction performance. Matsuo et al. [13] demonstrated that random forest showed good performance for poor outcome prediction at discharge and ridge regression for in-hospital mortality prediction in TBI, both of which achieved an accuracy of almost 0.9. Based on the feature selection method, age and GCS appeared to be the most important predictors for both poor outcome and mortality in their study, which was consistent with our findings. A total of 232 patients with TBI were included and separated into training data and test data, which was comparable to our samples of 230 patients. The prediction of mortality was better than our results, which were 0.886 accuracy and 0.875 AUC on the testing data. The difference in performance is mainly due to the prediction of death at different times, and there is no doubt that long-term mortality is harder to predict than in-hospital mortality. Rughani et al. [33] used an artificial neural network to predict the in-hospital survival of TBI patients, which achieved an accuracy of 0.878 and an AUC of 0.860. They included 11 variables in the model: age, sex, total GCS score, individual components of the GCS score at the scene of injury and emergency department, and first systolic blood pressure. Nonetheless, some vital parameters, such as biochemical tests, CT scan characteristics, and neurologic worsening conditions, were absent from their model. Although one study predicted 18-month mortality in severe TBI after DC using the IMPACT prognostic model [19], whose AUC was 0.77, our random tree prediction model achieved AUCs of 0.998 and 0.830 on the testing and training data, respectively. This suggests that machine learning models perform better in outcome prediction than traditional logistic regression models. At present, machine learning algorithms have been increasingly used in the prognosis of TBI [13, 34, 35], and they enable us to optimize the treatment strategy and provide better daily care.

### Limitations

There are several limitations to our study. First, it was a single-center, retrospective, and nonrandomized study, so selection bias may exist. Second, we did not include ICP data in this study because ICP monitoring was not performed on every patient. Thus, we could not include this vital variable to avoid apparent selection bias. Another limitation is that some information about the patient after discharge, such as rehabilitation therapy, was absent, but this provides an opportunity for prospective research to analyze this variable. Finally, our prognostic model was mainly targeted at TBI patients undergoing DC, so the performance of our model may decrease when it is applied to all TBI patients.

## Conclusions

Our findings confirm that older age, lower GCS, higher D-dimer, coagulopathy, hypotension, and completely effaced basal cisterns were associated with 1-year mortality. The random forest model showed relatively good predictive performance for 1-year mortality, which achieved an overall accuracy of 0.810, sensitivity of 0.833, specificity of 0.800, and AUC of 0.830 on the testing data. Our results indicated that machine learning achieved good performance for TBI outcomes. By virtue of machine learning with more accurate prediction performance, we can provide TBI patients with better precision medicine and care directions.

## Data Availability

Please contact the authors for data requests.

## Abbreviations

DC: Decompressive craniectomy
TBI: Traumatic brain injury
AUCs: Area under the receiver operating characteristic curves
GCS: Glasgow Coma Score
ICP: Intracranial pressure
